# The STELLAR trial: a phase II/III randomized trial of high-dose, intermittent sunitinib in patients with recurrent glioblastoma

**DOI:** 10.1093/braincomms/fcae241

**Published:** 2024-07-30

**Authors:** Jorien B E Janssen, Cyrillo G Brahm, Chantal M L Driessen, Janine Nuver, Mariette Labots, Mathilde C M Kouwenhoven, Esther Sanchez Aliaga, Roelien H Enting, Jan Cees de Groot, Annemiek M E Walenkamp, Myra E van Linde, Henk M W Verheul

**Affiliations:** Department of Medical Oncology, Research Institute for Medical Innovation, Radboud University Medical Center, 6525 GA, Nijmegen, The Netherlands; Department of Medical Oncology, Cancer Center Amsterdam, Vrije Universiteit Amsterdam, Amsterdam UMC, 1081 HV, Amsterdam, The Netherlands; Department of Medical Oncology, Research Institute for Medical Innovation, Radboud University Medical Center, 6525 GA, Nijmegen, The Netherlands; Department of Medical Oncology, University Medical Center Groningen, University Groningen, 9713 GZ, Groningen, The Netherlands; Department of Medical Oncology, Cancer Center Amsterdam, Vrije Universiteit Amsterdam, Amsterdam UMC, 1081 HV, Amsterdam, The Netherlands; Department of Neurology, Cancer Center Amsterdam, Vrije Universiteit Amsterdam, Amsterdam UMC, 1081 HV, Amsterdam, The Netherlands; Department of Radiology and Nuclear Medicine, Vrije Universiteit Amsterdam, Amsterdam UMC, 1081 HV, Amsterdam, The Netherlands; Department of Neurology, University Medical Center Groningen, University Groningen, 9713 GZ, Groningen, The Netherlands; Department of Radiology, Medical Imaging Center, University Medical Center Groningen, University Groningen, 9713 GZ, Groningen, The Netherlands; Department of Medical Oncology, University Medical Center Groningen, University Groningen, 9713 GZ, Groningen, The Netherlands; Department of Medical Oncology, Cancer Center Amsterdam, Vrije Universiteit Amsterdam, Amsterdam UMC, 1081 HV, Amsterdam, The Netherlands; Department of Medical Oncology, Research Institute for Medical Innovation, Radboud University Medical Center, 6525 GA, Nijmegen, The Netherlands; Department of Medical Oncology, Erasmus University Medical Center, Cancer Institute, 3015 GD, Rotterdam, The Netherlands

**Keywords:** glioblastoma, tyrosine kinase inhibitors, sunitinib, high-dose, lomustine

## Abstract

Previously, the tyrosine kinase inhibitor sunitinib failed to show clinical benefit in patients with recurrent glioblastoma. Low intratumoural sunitinib accumulation in glioblastoma patients was reported as a possible explanation for the lack of therapeutic benefit. We designed a randomized phase II/III trial to evaluate whether a high-dose intermittent sunitinib schedule, aimed to increase intratumoural drug concentrations, would result in improved clinical benefit compared to standard treatment with lomustine. Patients with recurrent glioblastoma were randomized 1:1 to high-dose intermittent sunitinib 300 mg once weekly (Q1W, part 1) or 700 mg once every two weeks (Q2W, part 2) or lomustine. The primary end-point was progression-free survival. Based on the pre-planned interim analysis, the trial was terminated for futility after including 26 and 29 patients in parts 1 and 2. Median progression-free survival of sunitinib 300 mg Q1W was 1.5 months (95% CI 1.4–1.7) compared to 1.5 months (95% CI 1.4–1.6) in the lomustine arm (*P* = 0.59). Median progression-free survival of sunitinib 700 mg Q2W was 1.4 months (95% CI 1.2–1.6) versus 1.6 months (95% CI 1.3–1.8) for lomustine (*P* = 0.70). Adverse events (≥grade 3) were observed in 25%, 21% and 31% of patients treated with sunitinib 300 mg Q1W, sunitinib 700 mg Q2W and lomustine, respectively (*P* = 0.92). To conclude, high-dose intermittent sunitinib treatment failed to improve the outcome of patients with recurrent glioblastoma when compared to standard lomustine therapy. Since lomustine remains a poor standard treatment strategy for glioblastoma, innovative treatment strategies are urgently needed.

## Teaser

The authors present the results of the STELLAR trial that compared the efficacy of two alternative high-dose intermittent sunitinib schedules against lomustine in 55 patients with recurrent glioblastoma. Therapy related toxicities of sunitinib were acceptable and comparable to lomustine. The trial was terminated for futility after the interim analysis.

## Graphical Abstract

**Graphical Abstract:**
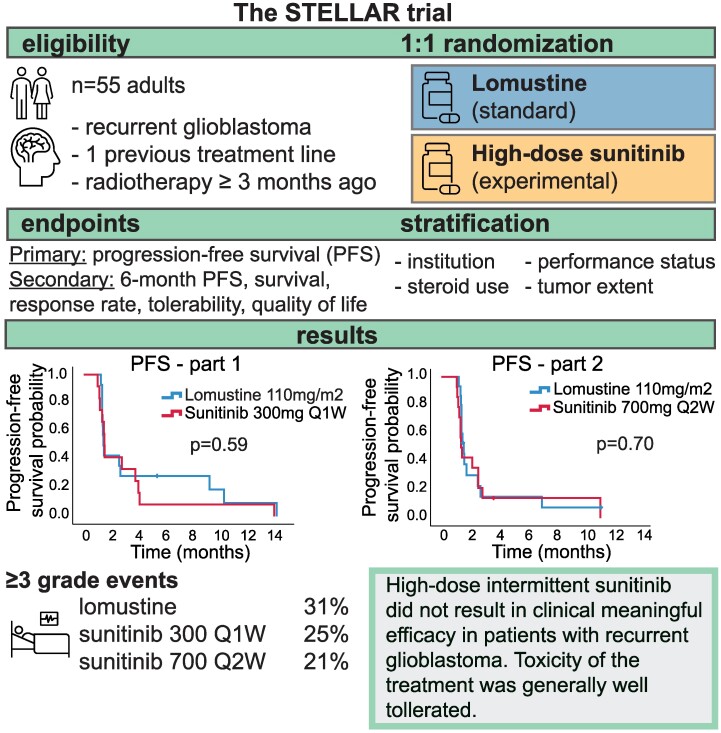

## Introduction

Despite increased knowledge of tumour biology and the introduction of targeted therapies such as tyrosine kinase inhibitors, proven effective in many solid and haematological tumours, prognosis for patients with glioblastoma remained grim over the past 10 years. Treatment according to the STUPP protocol, consisting of maximal surgical resection, followed by radiotherapy with concurrent temozolomide and six cycles of adjuvant temozolomide is associated with a median overall survival (OS) of 15–18 months.^1-4^ At the time of recurrence, which is almost inevitable, a clinically meaningful standard treatment is lacking.^5,6^ Treatment options in the recurrent setting are re-irradiation or re-resection (only for selected patients), systemic therapy with lomustine, study participation or best supportive care (BSC).^5,7^ Lomustine, an alkylating agent, is often prescribed to patients with recurrent disease after first-line treatment with chemoradiation.^8-10^ As single agent, lomustine resulted in low objective response rates, a 6-month PFS rate of 10–20% and median progression-free survival (PFS) of two months, approximately.^8-13^ Therefore, more effective treatment strategies for recurrent disease are urgently needed to improve the outlook of patients with recurrent glioblastoma.

Sunitinib is a multi-targeted tyrosine kinase inhibitor, most often applied in a dose of 50 mg once daily with incorporated recovery intervals. Originally it was developed as an inhibitor of the vascular endothelial growth factor receptor (VEGFR), platelet-derived growth factor receptor (PDGFR), stem cell factor receptor c-KIT, FLT3, CSF-1R and RET kinases. While sunitinib inhibits these targets at low concentrations, it also has affinity for multiple other kinases at higher drug concentrations.^14,15^ Since many of its targets are overexpressed or amplified in glioblastoma, sunitinib is a potentially interesting treatment.^16-18^ However, standard-dosed sunitinib had insufficient anti-tumour activity in patients with recurrent high-grade glioma.^19-22^ As a potential clinical resistance mechanism, low intratumoural drug concentrations are achieved upon standard treatment, presumably due to the blood–brain barrier, which hinders adequate intracerebral drug penetration.^23,24^ In a pilot study, median tumour concentration after two weeks of treatment with sunitinib 50 mg once daily in patients with newly diagnosed glioblastoma was 1.9 µmol/L (range 1.0–3.4).^25^ Despite the 10-fold higher tumour concentrations compared to median plasma concentrations, these sunitinib concentrations were evidently lower than its IC50 values in GBM cell lines *in vitro* and lower compared to sunitinib tumour concentrations in other solid malignancies.^26^

Hypothesizing that higher tumour sunitinib concentrations may improve its benefit by inhibiting relevant off-target kinase activity, we previously developed a high-dose, intermittent sunitinib treatment schedule. The maximum tolerated dose was established at 300 mg once weekly (Q1W) and 700 mg once every two weeks (Q2W) in a phase I clinical trial in patients with treatment refractory solid tumours. This alternative strategy was feasible and safe with a toxicity profile comparable to the standard dosing schedule while high plasma peak concentrations were reached.^27^ Intratumoural concentrations achieved with 300 mg Q1W and 700 mg Q2W high-dose intermittent sunitinib treatment were 19-fold and 37-fold higher compared to plasma concentrations.^28^ Importantly, tumour concentrations were 2–5 times higher 2 days after oral administration compared to those reached with the regular dosing schedule.^26,28^ In addition, preliminary results indicated efficacy and higher intratumoural drug concentrations were associated with longer PFS and OS in this heavily pre-treated phase I population.^28^ Based on these promising results, we tested short exposure of high-dose sunitinib on glioblastoma cell lines. Subsequently, we designed a phase II/III randomized clinical trial to study the efficacy of high-dose intermittent sunitinib compared to standard of care with lomustine in patients with recurrent glioblastoma.

## Materials and methods

### *In vitro* assessment of sunitinib activity

For the *in vitro* high-dose, short-term exposure experiments, four glioblastoma cell lines were used: U-87MG, U-251MG, U-138MG and T98G. Cell lines were exposed to different concentrations of sunitinib 5 µM, 10 µM and 20 µM as described previously.^29^ Read-out of cell viability was performed after 144 h using an MTT assay (see Supplementary methods and Rovithi *et al*.^29^ and Gotink *et al*.^30^ for full methods). Statistical analysis was performed in GraphPad Prism 10.1.2 using one-way ANOVA followed by Tukey’s *post hoc* test.

### Study design

A randomized, open-label, multicentre, phase II/III trial was conducted in patients with recurrent glioblastoma in three centres in The Netherlands. Eligible patients were randomized 1:1 with variable block size (4, 6 or 8) in Castor EDC to treatment with high-dose intermittent sunitinib or lomustine.^31^ Fifty patients per arm were required to detect clinical benefit from the experimental arm, while after 25% of patients inclusion, an interim analysis was planned. Patients were stratified according to the treatment centre, the WHO performance status (0 versus ≥1), the use of steroids and the extent of their disease (unifocal versus multifocal). The study was conducted in accordance with the Declaration of Helsinki and Good Clinical Practice guidelines. The Medical Research Ethics Committee of Amsterdam UMC, location VUmc, Amsterdam, and the local institutional review board of the participating centres approved the clinical trial protocol. Written informed consent was obtained from all patients before study inclusion. The trial was registered on ClinicalTrials.gov (https://clinicaltrials.gov/; NCT03025893).

### Patient eligibility

Eligible patients were adults with histologically confirmed *de novo* or secondary glioblastoma with first progression/recurrence, who received one prior line of chemotherapy (radiotherapy with concomitant and adjuvant temozolomide is considered as one line). Furthermore, patients should have completed radiotherapy at least three months before randomization and chemotherapy at least four weeks before randomization. Prior surgery for recurrence was allowed if the recurrence was histopathologically confirmed. Karnofsky performance status of ≥70% and adequate haematological, renal and hepatic functions were required. Main exclusion criteria included any significant uncontrolled concomitant disease, second primary malignancy in the past five years, prior radiotherapy in the abdomen, lungs, or >3 vertebrae in the spine, poorly controlled hypertension (>160/95 mmHg) and the use of full dose anticoagulants or strong hepatic enzyme-inducing anti-epileptic drugs (EIAEDs). Patients using EIAEDs had to switch to an anti-epileptic drug not interacting with cytochrome P450 before starting study treatment. Before trial participation an MRI-cerebrum should rule out intratumoural haemorrhage, except for stable postoperative grade 1 haemorrhage. Full in- and exclusion criteria are available in the Supplementary methods. Patients were included based on the 2016 WHO classification of tumours of the Central Nervous System.

### Treatment

Patients in the control arm were treated with lomustine once every six weeks at a dose of 110 mg/m^2^ (maximum 200 mg). Patients in the experimental arm received high-dose intermittent sunitinib at a dose of 300 mg Q1W. Treatment was continued until disease progression, intolerable toxicity, withdrawal of consent, or any other medical reason according to the treating physician. Patients in the sunitinib arm could cross-over to lomustine after disease progression occurred.

After the planned interim analysis, the protocol was amended to change the dose of sunitinib from 300 mg Q1W (part 1) to 700 mg Q2W (part 2). At the start of the trial, the dose of 300 mg Q1W was selected because the safety of the 700 mg schedule was under evaluation. After the treatment of 12 patients with high-dose intermittent sunitinib in part 1, no major safety issues were observed, particularly no intracranial bleeding. As we anticipated higher intratumoural concentrations with the 700 mg Q2W schedule and, thereby, an improved treatment benefit, the protocol was amended to change the dosing schedule of sunitinib from 300 mg Q1W to 700 mg Q2W. Subsequently, the trial was restarted with inclusion of 100 patients with a pre-planned interim analysis after the inclusion of 25% of the patients. The results from the interim analysis of part 1 and part 2 are reported here.

### Study procedures

Tumour assessment was performed with an MRI-scan at baseline and subsequently every 6 weeks, which were evaluated according to the Response Assessment in Neuro-Oncology (RANO) criteria and centrally reviewed in part 2.^32^ Toxicity was scored according to the Common Terminology Criteria for Adverse Events (CTCAE), version 4.03.^33^ Patients were asked to complete Quality of Life (QoL) questionnaires every six weeks during treatment, consisting of the European Organization for Research and Treatment of Cancer (EORTC) QLQ-C30 and QLQ-BN20.^34-36^

### Study outcome

The primary end-point was the median PFS, assessed using RANO criteria. PFS was defined as the date of randomization to the date of progressive disease or death. If evidence of PD was disputable, treatment could be continued until the next assessment, but if PD was confirmed at the next follow-up, the earlier date was used as the date of progression. Secondary end-points included the six-month progression-free survival (PFS6), OS, objective radiological response rate (according to the RANO criteria), toxicity and side effects of the treatment, QoL and steroid use, the potential value of blood biomarkers and the influence of O^6^-methylguanine-DNA methyl-transferase (*MGMT*) promotor methylation status on sunitinib response. OS was defined as the date from randomization until the date of death from any cause. Patients who were progression-free and/or alive at the date of the analysis and patients who were lost to follow-up were censored at the date of the last follow-up visit.

### Statistical analysis

Median PFS and PFS-6 rates, as reported in previous trials with recurrent glioblastoma, are 1.6–2.7 months and 19%, respectively.^9,11^ For the sample size calculation, we assumed equal accrual over 24 months, with a minimum follow-up duration of 12 months. To detect an increase in median PFS from 2.5 months with lomustine to 5.5 months with high-dose intermittent sunitinib (corresponding with a HR of <0.45), 50 patients per arm were needed (total of 100 patients) to yield a power of 95% (assuming two-sided testing at a significance level of 5%). After 25% of patients were included, a pre-planned interim analysis was performed for futility. In case the number of events in the high-dose sunitinib arm exceeds the number of events in the lomustine arm, the study was stopped for futility. All evaluable patients, defined as those completing at minimum six weeks of treatment, were included in the analysis. Median PFS, median OS and PFS-6 were estimated using the Kaplan–Meier method. PFS and OS were compared between groups using the log-rank test. Cox regression analysis was used to estimate hazard ratio for treatment with high-dose intermittent sunitinib compared to lomustine. Log minus log curves were used prior to Cox regression to verify that proportional hazard assumptions were not violated. Descriptive statistics were used for reporting baseline characteristics and adverse events. Linear mixed model analysis was used to analyse QoL end-points. Patients were included in the QoL analysis if at least a baseline questionnaire and one follow-up questionnaire were available. Five scales were selected before analysis (summary score, physical functioning, visual disorders, motor dysfunction and communication deficit), which were considered more relevant than the selection in the protocol. EORTC guidelines were used for the scoring of the QoL questionnaires.^37^ Raw scores were calculated as the mean of items part of that particular scale and were transformed linearly into scores ranging from 0 to 100. Higher scores for the summary score and functional scales represent better HRQoL and functioning, respectively, while higher scores on symptom scales represent more presence of symptoms. Kaplan–Meier method and Cox regression analysis were applied to investigate the effect of *MGMT* status on OS and PFS as exploratory end-points. Comparison of occurrence of grade ≥ 3 adverse events in treatment groups was performed using Fisher’s Exact test. Statistical analyses were performed using IBM SPSS (version 27) software.

## Results

### Sensitivity of GBM cell lines to high-dose sunitinib exposure

*In vitro,* we observed that high-dose, short-term sunitinib exposure inhibited the proliferation of all four glioblastoma cell lines with increased efficacy at higher doses (Supplementary Fig. 1). After 9 h exposure, proliferation was significantly inhibited in two out of four cell lines (20 μM versus 5 μM, *P* < 0.05). After 24 h exposure, proliferation was significantly inhibited in all four cell lines (20 μM versus 5 μM, *P* < 0.05). A difference in sensitivity towards high-dose, short-term sunitinib exposure was observed between the four cell lines. Cell death was observed at higher concentrations, compared to the start of treatment, depicted as negative values.

### Patients

**Figure 1:**
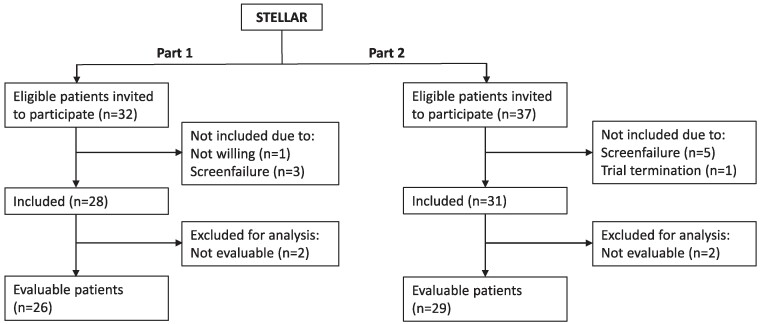
**CONSORT diagram of patients invited to participate and included in the STELLAR trial.** Overview of patient enrolment and availability for analysis. All evaluable patients, defined as those completing at minimum six weeks of treatment, were included in the analysis.

In part 1, 32 patients were invited to participate between September 2018 and November 2019. In part 2, 37 patients were invited to participate between September 2020 and March 2022. Twenty-six of 28 included patients in part 1 and 29 of 31 included patients in part 2 were evaluable according to the protocol (Consort Diagram in Fig. 1). The baseline characteristics of the evaluable patients are displayed in Table 1.

**Table 1:** Patient characteristics

	Part 1		Part 2	
Variables	Sunitinib 300 mg Q1W (*n* = 12)	Lomustine (*n* = 14)	Sunitinib 700 mg Q2W (*n* = 14)	Lomustine (*n* = 15)
Median age—years (range)	50 (34–63)	53 (30–71)	60 (40–77)	56 (39–68)
Sex—no (%)				
Male	11 (92%)	9 (64%)	9 (64%)	10 (67%)
Female	1 (8%)	5 (36%)	5 (36%)	5 (33%)
WHO status—no (%)				
0	6 (50%)	3 (21%)	3 (21%)	4 (27%)
≥1	6 (50%)	11 (79%)	11 (79%)	11 (73%)
Tumour extent—no (%)				
Unifocal	10 (83%)	10 (71%)	8 (57%)	8 (53%)
Multifocal	2 (17%)	4 (29%)	6 (43%)	7 (47%)
MGMT promoter status—no (%)				
Unmethylated	5 (42%)	8 (57%)	6 (43%)	10 (67%)
Methylated	5 (42%)	6 (43%)	6 (43%)	3 (20%)
Unknown	2 (17%)	0 (0%)	2 (14%)	2 (13%)
Steroid use—no (%)				
Yes	3 (25%)	9 (64%)	9 (64%)	8 (53%)
No	9 (75%)	5 (36%)	5 (36%)	7 (47%)
IDH				
Wild-type	9 (75%)	13 (93%)	13 (93%)	14 (93%)
Mutant	2 (17%)	1 (7%)	1 (7%)	0 (0%)
Unknown	1 (8%)	0 (0%)	0 (0%)	1 (7%)
First-line treatment				
Resection—no (%)				
Yes	11 (92%)	14 (100%)	11 (79%)	13 (87%)
No	1 (8%)	0 (0%)	3 (21%)	2 (13%)
Radiotherapy (RTx)—no (%)				
RTx + concomitant TMZ	11 (92%)	14 (100%)	12 (86%)	14 (93%)
RTx alone	1 (8%)	0 (0%)	1 (7%)	0 (0%)
Only chemotherapy	0 (0%)	0 (0%)	1 (7%)	1 (7%)
TMZ—no (%)				
<6 cycles	4 (33%)	6 (43%)	5 (36%)	4 (27%)
6 cycles	7 (58%)	7 (50%)	6 (43%)	10 (67%)
>6 cycles	1 (8%)	1 (7%)	3 (21%)	1 (7%)

Q1W, weekly; Q2W, bi-weekly; MGMT, O^6^-methylguanine-DNA methyl-transferase; TMZ, temozolomide.

### Efficacy

**Figure 2:**
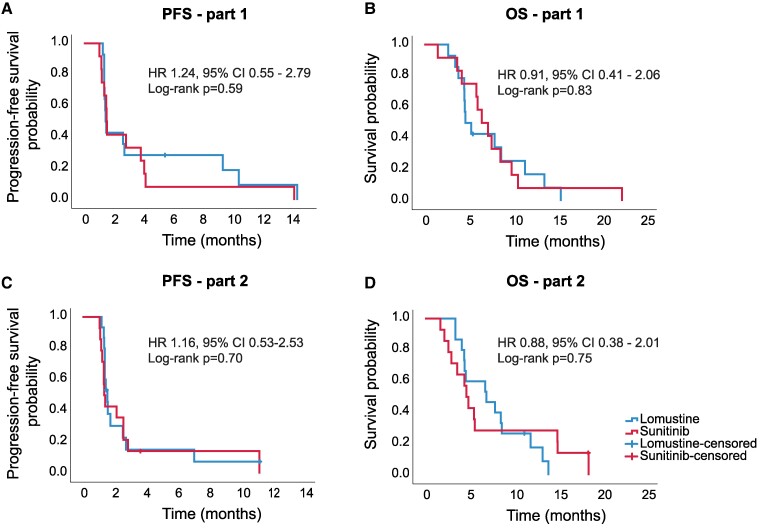
**Progression-free survival and overall survival.** Progression-free survival (**A**) and overall survival (**B**) in part 1. Part 1: lomustine *n* = 14, high-dose intermittent sunitinib *n* = 12. Progression-free survival (**C**) and overall survival (**D**) in part 2. Part 2: lomustine *n* = 15, high-dose intermittent sunitinib *n* = 14. PFS, progression-free survival; HR, hazard ratio; OS, overall survival.

The data cut-off for efficacy was on 1 February 2023. The PFS of all patients is presented in Fig. 2. In part 1, mPFS of sunitinib 300 mg Q1W was 1.5 months (95% CI 1.4–1.7) compared to 1.5 months (95% CI 1.4–1.6) for lomustine (*P* = 0.59). In part 2, mPFS of sunitinib 700 mg Q2W was 1.4 months (95% CI 1.2–1.6) versus 1.6 months (95% CI 1.3–1.8) for lomustine (*P* = 0.70). The hazard ratio for progression or death was 1.24 (95% CI 0.55–2.79) and 1.16 (95% CI 0.53–2.53) in parts 1 and 2, respectively. No significant differences were observed between the median PFS of lomustine and the two cohorts of high-dose intermittent sunitinib. Six-month PFS rates for sunitinib and lomustine in part 1 were 8% (95% CI 0–24%) and 29% (95% CI 5–52%), respectively, and 14% (95% CI 0–33%) and 15% (95% CI 0–34%) for part 2. In part 1, one partial response was observed in a patient treated with sunitinib 300 mg Q1W [objective response rate (ORR) 8%] and in one patient treated with lomustine (ORR 7%). In part 2, the best radiological response was stable disease. Radiological response rates are presented in Supplementary Table 1.

Overall survival was comparable for the two cohorts of high-dose intermittent sunitinib and lomustine (Fig. 2). In part 1, mOS for sunitinib 300 mg Q1W was 6.5 months (95% CI 4.5–8.5) compared to 4.7 (95% CI 3.3–6.0) in the lomustine arm (*P* = 0.83) with a hazard ratio for death of 0.91 (95% CI 0.41–2.06). In part 2, mOS for sunitinib 700 mg Q2W was 4.7 months (95% CI 3.9–5.5) compared to 7.0 months (95% CI 2.8–11.2) in the lomustine arm (*P* = 0.75). Hazard ratio for death was 0.88 (95% CI 0.38–2.01).

After the inclusion of 26 patients in part 2 of the STELLAR trial, a pre-planned interim analysis was performed for futility. Based on the results, we calculated the chance to detect an improved PFS for treatment with high-dose intermittent sunitinib when we would complete the study inclusion (*n* = 100). The estimated chance to demonstrate superiority of high-dose intermittent sunitinib over lomustine was extremely low (<5%) and considered unrealistic. After consultation with the Medical Ethics Board, it was decided to terminate the study to prevent possible safety risks and burdens from a non-effective experimental treatment.

### Safety

Grade 1 or 2 related adverse events (AE) that occurred in ≥10% in any treatment group, and all grade 3 or 4 related adverse events are reported in Table 2. In the sunitinib group, most frequently reported AEs were thrombocytopaenia (75%), fatigue (58%) and leukopaenia (58%) for patients treated with 300 mg Q1W and thrombocytopaenia (57%), fatigue (50%) and diarrhoea (43%) for patients treated with 700 mg Q2W. Most adverse events were manageable with standard supportive interventions. Diarrhoea, nausea and vomiting caused by sunitinib occurred mainly in the first 2 days after sunitinib intake. In part 1, one patient in the sunitinib arm required a dose reduction due to palmar–plantar erythrodysesthesia syndrome. One patient in the sunitinib arm required surgical drainage and antibiotic treatment for grade 4 septic bursitis. In part 2, four patients required a dose reduction for skin rash (sunitinib), grade 3 liver test abnormalities (sunitinib), leukopaenia (lomustine) and thrombocytopaenia (lomustine). The most frequently reported AEs in the lomustine group were thrombopaenia (48%), fatigue (45%) and leukopaenia (34%). Dose delays due to treatment-related AEs were required in the sunitinib and lomustine arm in 42% and 21% of patients in part 1 and 14% and 13% of patients in part 2. In two patients of the sunitinib arm in part 2 (14%), intolerable toxicity was the reason for trial discontinuation, while in part 1, no patient discontinued due to toxicity. Two patients (7%) treated with lomustine required one or multiple platelet transfusions. One patient in the sunitinib arm had an intratumoural haemorrhage at the time of rapid progression of the tumour with the occurrence of new leptomeningeal metastases, which was attributed to progressive disease. No deaths were considered to be related to sunitinib or lomustine.

**Table 2:** Adverse events

	Lomustine (*n* = 29)		Sunitinib 300 mg Q1W (*n* = 12)		Sunitinib 700 mg Q2W (*n* = 14)	
	Grades 1–2	Grades 3–4	Grades 1–2	Grades 3–4	Grades 1–2	Grades 3–4
Clinical adverse events						
Blurred vision	0 (0)		0 (0)		2 (14)	
Diarrhoea	1 (3)		2 (17)		6 (43)	
Dysgeusia	0 (0)		3 (25)		2 (14)	
Fatigue	12 (41)	1 (3)	7 (58)		7 (50)	
Flu like symptoms	0 (0)		3 (25)		2 (14)	
Headache	1 (3)		2 (17)		2 (14)	1 (7)
Hypertension	0 (0)		2 (17)		1 (7)	1 (7)
Hypothyroidism	0 (0)		2 (17)		0 (0)	
Mucositis oral	0 (0)		1 (8)		3 (21)	1 (7)
Muscle weakness lower limb	1 (3)		0 (0)		0 (0)	1 (7)
Musculoskeletal disorders (e.g. arthralgia and myalgia)	0 (0)		3 (25)		1 (7)	
Nausea	7 (24)		3 (25)		4 (29)	
Oral pain	0 (0)		0 (0)		2 (14)	
Palmar–plantar erythrodysesthesia syndrome	0 (0)		3 (25)		0 (0)	
Rash	1 (3)		5 (42)		3 (21)	
Septic bursitis	0 (0)		0 (0)	1 (8)	0 (0)	
Skin discolouration	0 (0)		3 (25)		4 (29)	
Syncope	0 (0)		0 (0)		0 (0)	1 (7)
Tooth infection	0 (0)		0 (0)	1 (8)	0 (0)	
Vertigo	0 (0)		0 (0)		2 (14)	
Vomiting	3 (10)		1 (8)		3 (21)	
Laboratory adverse events—haematologic						
Anaemia	8 (28)		4 (33)		1 (7)	
Lymphocyte count decreased	4 (14)	2 (7)	4 (33)	1 (8)	1 (7)	
Neutrophil count decreased	3 (10)	2 (7)	4 (33)	1 (8)	1 (7)	
Platelet count decreased	10 (34)	4 (14)	9 (75)		8 (57)	
White blood cell decreased	8 (28)	2 (7)	7 (58)		4 (29)	1 (7)
Laboratory adverse events—liver						
Alanine aminotransferase increased	3 (10)		4 (33)		2 (14)	1 (7)
Alkaline phosphatase increased	0 (0)		2 (17)		1 (7)	
Aspartate aminotransferase increased	2 (7)		2 (17)		2 (14)	
GGT increased	0 (0)		1 (8)		1 (7)	1 (7)

All treatment-related adverse events are reported. Grade 1–2 adverse events are reported if occurring in ≥10% of patients in one of three treatment groups, all grade 3–4 events are reported. Adverse events for lomustine from part 1 and part 2 are reported together. Q1W, weekly; Q2W, bi-weekly; GGT, gamma-glutamyltransferase.

### Quality of life

QoL data from part 1 and part 2 were analysed in one combined analysis to compare QoL between patients treated with high-dose intermittent sunitinib and patients treated with lomustine. We restricted the analysis to the first four follow-up moments since after that the number of questionnaires was too limited to draw any conclusions. For 65% of patients, at least the baseline and one follow-up questionnaire were available. QoL did not differ between treatment groups on the parameters’ summary score, physical functioning, visual disorders, motor dysfunction and communication deficit between patients treated with high-dose intermittent sunitinib and patients treated with lomustine (estimated marginal means shown in Supplementary Table 2).

### Efficacy of lomustine in the two control arms

**Figure 3:**
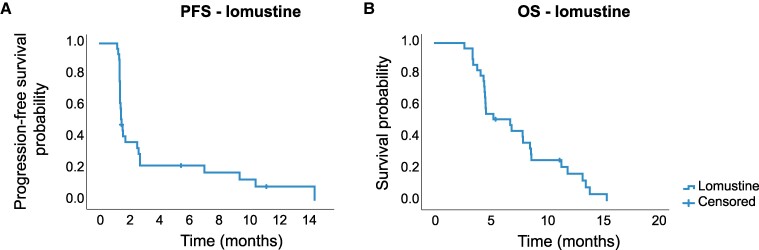
**Efficacy lomustine cohort.** (**A**) Progression-free survival of patients treated with lomustine (*n* = 29). (**B**) Overall survival of patients treated with lomustine (*n* = 29). PFS, progression-free survival; OS, overall survival.

The efficacy of lomustine in the pooled data of 29 patients in the two control arms revealed a 6-month PFS rate of 22% (95% CI 7–38%), mPFS of 1.5 months (95% CI 1.3–1.7) and mOS of 6.8 months (95% CI 3.0–10.7) (Fig. 3). PFS and OS were comparable in the two independent lomustine cohorts with a hazard ratio of 0.82 (95% CI 0.37–1.81; *P* = 0.62) and 0.97 (95% CI 0.45–2.11; *P* = 0.94), respectively, in part 1 as compared to part 2.

### Molecular markers

**Figure 4:**
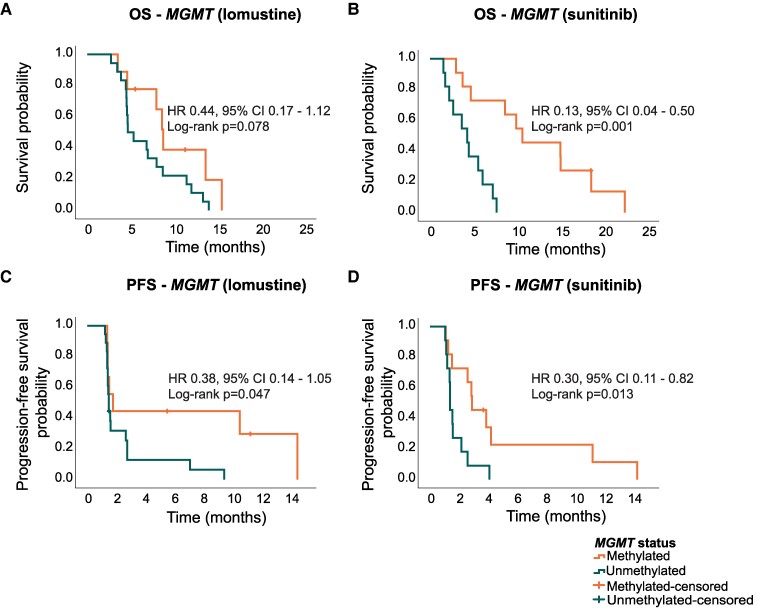
***MGMT* status in relation to PFS and OS.** (**A**) *MGMT* status in relation to OS in patients treated with lomustine (*n* = 27). (**B**) *MGMT* status in relation to OS in patients treated with sunitinib (*n* = 22). (**C**) *MGMT* status in relation to PFS in patients treated with lomustine (*n* = 27). (**D**) *MGMT* status in relation to PFS in patients treated with sunitinib (*n* = 22). In the lomustine arm, *n* = 9 had methylated *MGMT* and *n* = 18 unmethylated *MGMT*. In the high-dose intermittent sunitinib arm, *n* = 11 had methylated *MGMT* and *n* = 11 unmethylated *MGMT*. *MGMT*, O^6^-methylguanine-DNA methyl-transferase; HR, hazard ratio; PFS, progression-free survival; OS, overall survival.

*MGMT* status was known in 49/55 cases (89%); 20 patients had a methylated *MGMT* promotor (41%). In both sunitinib arms, 11/22 patients had methylated *MGMT* promotor (50%) compared to 9/27 (33%) in the lomustine groups. Consistent with previous findings, *MGMT* promotor methylation was associated with improved survival [mOS 9.9 months (95% CI 7.1–12.7) versus 4.6 months (95% CI 4.4–4.9; *P* < 0.001)] (Supplementary Fig. 2). For sunitinib, patients with methylated versus unmethylated *MGMT* status had significantly longer median PFS [2.9 months (95% CI 1.6–4.2) versus 1.4 months (95% CI 1.2–1.6; *P* = 0.013)] and median OS [10.6 months (95% CI 3.8–17.5) versus 4.3 (95% CI 2.3–6.2; *P* = 0.001)]. For lomustine, these differences were 1.8 (95% CI 1.0–2.5) versus 1.5 months (95% CI 1.4–1.5; *P* = 0.047) months for PFS and 8.7 (95% CI 7.7–9.8) versus 4.7 (95% CI 3.2–6.1) months for OS (*P* = 0.078) (Fig. 4).

An additional exploratory analysis was performed that excluded patients with an *IDH* mutation. *IDH* mutation status was known for 53/55 patients. In total, four patients had a *IDH* mutation. In this analysis, median PFS was 1.4 months (95% CI 1.3–1.6) for patients treated with high-dose intermittent sunitinib and 1.5 months (95% CI 1.3–1.6) for patients treated with lomustine. Median OS was 5.6 months (95% CI 4.1–7.0) for high-dose intermittent sunitinib versus 5.3 months (95% CI 2.4–8.2) for lomustine (Supplementary Fig. 3).

## Discussion

In this phase II/III clinical trial, we evaluated the efficacy of high-dose intermittent sunitinib for patients with recurrent glioblastoma. Previously, in a small group of patients with glioblastoma, we reported that intratumoural drug concentrations after two weeks of regular dose sunitinib treatment were lower compared to tumour concentrations in the metastases of other tumour types.^25,26^ These data suggest that sunitinib drug delivery is hampered by the blood–brain barrier, which is believed not to be completely disrupted.^23,38^ In addition, drug efflux transporters on endothelial cells may interfere with drug penetration.^23,39,40^ Based on the two and five times higher tumour concentrations of the high-dosed schedules of 300 mg Q1W and 700 mg Q2W compared to standard-dosed sunitinib, we hypothesized that with this alternative high-dose treatment strategy higher intratumoural drug concentrations would be achieved, sufficient to inhibit designated targets of sunitinib as well as potential off-target, lower affinity kinases. However, in this phase II/III clinical trial, high-dose intermittent sunitinib failed to improve clinical benefit over lomustine for patients with recurrent glioblastoma at the pre-planned interim analyses. Consequently, the STELLAR study was terminated due to futility. The efficacy results obtained in this cohort are comparable to those observed with standard-dosed sunitinib in patients with recurrent glioblastoma. The mPFS in two of these studies were 1.6 months and 1.4 months, respectively, and no partial responses were observed.^19,20^ A limitation of the trial is the lack of selection based on *IDH* mutation status of the tumours. Therefore, we performed an additional exploratory analysis of *IDH* mutation status that identified four patients with an *IDH* mutation. A sub-analysis with exclusion of these patients did not significantly alter our results. Additionally, there was a subtle difference in steroid use between groups in part 1, although steroid use was one of the stratification factors in the trial. This is due to the block randomization ensuring a balance in stratification factors based on the expected total number of included patients without taking into account the sample size of the planned interim analysis. However, steroid use was not independently associated with either PFS (P = 0.74) or OS (*P* = 0.25) in our cohort. Therefore, we did not correct for this difference.

The reasons for this lack of clinical activity for high-dose intermittent sunitinib are unknown but may be attributed to several factors. Firstly, it should be considered that the participating patients in this trial had a poor prognosis indicated by recurrence of disease mostly short after completion of adjuvant temozolomide treatment or even during adjuvant therapy and patients were not selected based on the molecular characteristics of their tumours. Furthermore, intratumoural sunitinib concentrations may still be insufficient to exert anti-tumour efficacy. While we were able to demonstrate that higher tumour drug concentrations in patients with refractory non-CNS solid tumours were associated with longer PFS and OS,^28^ we were not able to determine intratumoural drug concentrations in this cohort of patients with recurrent glioblastoma. Intermittent dosing schedules exceeding 700 mg Q2W to potentially achieve even higher intratumoural concentrations are not feasible due to expected dose-limiting toxicities.^27^ Another potential explanation is that the biological characteristics of glioblastoma tumours determine their resistance to sunitinib. Since reported IC50 values for glioblastoma cell lines are higher (3.0–8.5 µmol/L) compared to IC50 values of cell lines from other tumour types (1.4–2.3 µmol/L), glioblastoma tumours may be inherently less sensitive to sunitinib.^25,30^ Further exploration of this intrinsic resistance is warranted on both the tumour cell and microenvironmental level.^41^ Additional factors to consider are which part of sunitinib (free, non-protein bound) can interact with its targets and its distribution within the tumour and inside the cell. Furthermore, immune escape and other escape mechanisms that are being exerted by the tumour cells, for example via activation of alternative pathways or through interaction with the micro-environment can influence treatment efficacy.^30,42-44^

Many tyrosine kinase inhibitors have been clinically evaluated for glioblastoma, while most provided no relevant benefit.^45^ Only in the phase II REGOMA study, the multikinase inhibitor regorafenib did improve overall survival compared to lomustine (mOS 7.4 versus 5.6 months, *P* = 0.0009). A follow-up phase III trial has to be performed to confirm these findings and assess whether the overall survival benefit is sufficient for clinical implementation.^13^ Additionally, in the subgroup of glioblastoma patients with a *BRAF* V600E mutation (∼3%), an objective response rate of 32% with durable clinical benefit was reported after treatment with dabrafenib–trametinib.^46^

The toxicity profile of high-dose intermittent sunitinib in this cohort was largely comparable to the toxicity observed in the phase I clinical trial.^27^ However, grade 1–2 thrombocytopaenia and leukopaenia (without need for intervention) occurred more frequently in the glioblastoma population compared to patients with solid tumours treated in the phase I trial. Treatment was well tolerated in most patients and toxicity was generally manageable.

While most studies suggest a predictive value of *MGMT* status for the response to alkylating agents in patients with recurrent glioblastoma,^47,48^ the difference in our cohort for lomustine treated patients was very modest. Due to the limited numbers in this sub-analysis, results should be carefully interpreted. In the sunitinib group, patients with methylated *MGMT* had a significantly longer PFS compared to patients with unmethylated *MGMT*. This finding contradicts *in vitro* results where MGMT expression (e.g. unmethylated status) leads to altered expression of receptor tyrosine kinases and significant inhibition of cell proliferation by the addition of sunitinib to the combination of TMZ and/or radiotherapy.^49^ These preclinical findings led to a phase II trial where sunitinib was added to standard first-line therapy in recurrent glioblastoma patients with unmethylated *MGMT*. While most patients only tolerated 12.5 mg of sunitinib daily in this study, a median PFS of 7.2 months and mOS of 15.0 months was observed in this single-arm study. Although the authors suggest that these results indicate a promising effect of sunitinib, the true benefit of this treatment strategy is at most limited as a first-line therapy in our opinion.^50^

Although lomustine has never been compared to BSC in a randomized trial, it is often considered to be the standard treatment for recurrent glioblastoma.^47^ The toxicity profile of lomustine in our study was comparable to previously observed toxicity.^8-13^ Clinically relevant toxicity consists of fatigue, nausea/vomiting, and haematological toxicity, which may require dose delays, dose reductions, or additional hospital visits. However, the limited efficacy of lomustine treatment in this trial—mPFS 1.5 months, mOS 6.8 months and six-month PFS rate of 22%—are in line with the disappointing efficacy described in the literature. Most patients do not derive any benefit from treatment with lomustine. Therefore, careful reconsideration whether lomustine treatment is of sufficient added benefit for patients with a glioblastoma is warranted when taking into consideration current guidelines such as the ESMO-Magnitude of Clinical Benefit Scale and the ASCO Value Framework Net Health Benefit Score.^51-53^ Potential biomarkers, such as *MGMT* status, might play a role in patient selection for treatment with lomustine but need formal proof.

To conclude, the STELLAR trial failed to demonstrate anti-tumour efficacy for two schedules of high-dose intermittent sunitinib and was therefore halted after the interim analyses. These clinical trial results require reconsidering the value of alternative high-dose scheduling of tyrosine kinase inhibitors to improve the outcome of patients with recurrent glioblastoma. Additionally, the results raise the question whether treatment with lomustine should be offered to patients with recurrent glioblastoma considering its limited clinical benefit and indicates that innovative treatment strategies are urgently needed.

## Supplementary Material

fcae241_Supplementary_Data

## Data Availability

Anonymized data collected in this clinical trial supporting the main findings of this study were deposited into the DANS easy repository, https://doi.org/10.17026/dans-x38-pz3s. To protect the privacy and confidentiality of the patients who participated in this study, clinical data are available in the repository under restricted access and can be made available upon reasonable request. All requests will be reviewed by the corresponding author and principal investigator.

## References

[1] Stupp R, Mason WP, van den Bent MJ, et al Radiotherapy plus concomitant and adjuvant temozolomide for glioblastoma. N Engl J Med. 2005;352(10):987–996. 15758009 10.1056/NEJMoa043330

[2] Wen PY, Weller M, Lee EQ, et al Glioblastoma in adults: A Society for Neuro-Oncology (SNO) and European Society of Neuro-Oncology (EANO) consensus review on current management and future directions. Neuro Oncol. 2020;22(8):1073–1113. 32328653 10.1093/neuonc/noaa106 PMC7594557

[3] Stupp R, Taillibert S, Kanner AA, et al Maintenance therapy with tumor-treating fields plus temozolomide vs temozolomide alone for glioblastoma: A randomized clinical trial. JAMA. 2015;314(23):2535–2543. 26670971 10.1001/jama.2015.16669

[4] Stupp R, Taillibert S, Kanner A, et al Effect of tumor-treating fields plus maintenance temozolomide vs maintenance temozolomide alone on survival in patients with glioblastoma: A randomized clinical trial. JAMA. 2017;318(23):2306–2316. 29260225 10.1001/jama.2017.18718 PMC5820703

[5] Seystahl K, Wick W, Weller M. Therapeutic options in recurrent glioblastoma—An update. Crit Rev Oncol Hematol. 2016;99:389–408. 26830009 10.1016/j.critrevonc.2016.01.018

[6] Stupp R, Hegi ME, Mason WP, et al Effects of radiotherapy with concomitant and adjuvant temozolomide versus radiotherapy alone on survival in glioblastoma in a randomised phase III study: 5-year analysis of the EORTC-NCIC trial. Lancet Oncol. 2009;10(5):459–466. 19269895 10.1016/S1470-2045(09)70025-7

[7] Weller M, van den Bent M, Preusser M, et al EANO guidelines on the diagnosis and treatment of diffuse gliomas of adulthood. Nat Rev Clin Oncol. 2021;18(3):170–186. 33293629 10.1038/s41571-020-00447-z PMC7904519

[8] Taal W, Oosterkamp HM, Walenkamp AME, et al Single-agent bevacizumab or lomustine versus a combination of bevacizumab plus lomustine in patients with recurrent glioblastoma (BELOB trial): A randomised controlled phase 2 trial. Lancet Oncol. 2014;15(9):943–953. 25035291 10.1016/S1470-2045(14)70314-6

[9] Wick W, Puduvalli VK, Chamberlain MC, et al Phase III study of enzastaurin compared with lomustine in the treatment of recurrent intracranial glioblastoma. J Clin Oncol. 2010;28(7):1168–1174. 20124186 10.1200/JCO.2009.23.2595 PMC2834468

[10] Wick W, Gorlia T, Bendszus M, et al Lomustine and bevacizumab in progressive glioblastoma. N Engl J Med. 2017;377(20):1954–1963. 29141164 10.1056/NEJMoa1707358

[11] Batchelor TT, Mulholland P, Neyns B, et al Phase III randomized trial comparing the efficacy of cediranib as monotherapy, and in combination with lomustine, versus lomustine alone in patients with recurrent glioblastoma. J Clin Oncol. 2013;31(26):3212–3218. 23940216 10.1200/JCO.2012.47.2464 PMC4021043

[12] Brandes AA, Carpentier AF, Kesari S, et al A phase II randomized study of galunisertib monotherapy or galunisertib plus lomustine compared with lomustine monotherapy in patients with recurrent glioblastoma. Neuro Oncol. 2016;18(8):1146–1156. 26902851 10.1093/neuonc/now009 PMC4933481

[13] Lombardi G, De Salvo GL, Brandes AA, et al Regorafenib compared with lomustine in patients with relapsed glioblastoma (REGOMA): A multicentre, open-label, randomised, controlled, phase 2 trial. Lancet Oncol. 2019;20(1):110–119. 30522967 10.1016/S1470-2045(18)30675-2

[14] Chow LQM, Eckhardt SG. Sunitinib: From rational design to clinical efficacy. J Clin Oncol. 2007;25(7):884–896. 17327610 10.1200/JCO.2006.06.3602

[15] Karaman MW, Herrgard S, Treiber DK, et al A quantitative analysis of kinase inhibitor selectivity. Nat Biotechnol. 2008;26(1):127–132. 18183025 10.1038/nbt1358

[16] Loureiro LVM, Neder L, Callegaro-Filho D, de Oliveira Koch L, Stavale JN, Malheiros SMF. The immunohistochemical landscape of the VEGF family and its receptors in glioblastomas. Surg Exp Pathol. 2020;3(1):9.

[17] Tilak M, Holborn J, New LA, Lalonde J, Jones N. Receptor tyrosine kinase signaling and targeting in glioblastoma multiforme. Int J Mol Sci. 2021;22(4):1831. 33673213 10.3390/ijms22041831 PMC7918566

[18] Joensuu H, Puputti M, Sihto H, Tynninen O, Nupponen NN. Amplification of genes encoding KIT, PDGFRalpha and VEGFR2 receptor tyrosine kinases is frequent in glioblastoma multiforme. J Pathol. 2005;207(2):224–231. 16021678 10.1002/path.1823

[19] Neyns B, Sadones J, Chaskis C, et al Phase II study of sunitinib malate in patients with recurrent high-grade glioma. J Neurooncol. 2011;103(3):491–501. 20872043 10.1007/s11060-010-0402-7

[20] Pan E, Yu D, Yue B, et al A prospective phase II single-institution trial of sunitinib for recurrent malignant glioma. J Neurooncol. 2012;110(1):111–118. 22832897 10.1007/s11060-012-0943-z PMC5735835

[21] Hutterer M, Nowosielski M, Haybaeck J, et al A single-arm phase II Austrian/German multicenter trial on continuous daily sunitinib in primary glioblastoma at first recurrence (SURGE 01-07). Neuro Oncol. 2013;16(1):92–102. 24311637 10.1093/neuonc/not161 PMC3870838

[22] Kreisl TN, Smith P, Sul J, et al Continuous daily sunitinib for recurrent glioblastoma. J Neurooncol. 2013;111(1):41–48. 23086433 10.1007/s11060-012-0988-z

[23] van Tellingen O, Yetkin-Arik B, de Gooijer MC, Wesseling P, Wurdinger T, de Vries HE. Overcoming the blood–brain tumor barrier for effective glioblastoma treatment. Drug Resist Updat. 2015;19:1–12. 25791797 10.1016/j.drup.2015.02.002

[24] Arvanitis CD, Ferraro GB, Jain RK. The blood–brain barrier and blood–tumour barrier in brain tumours and metastases. Nat Rev Cancer. 2020;20(1):26–41. 31601988 10.1038/s41568-019-0205-x PMC8246629

[25] van Linde ME, Labots M, Brahm CG, et al Tumor drug concentration and phosphoproteomic profiles after two weeks of treatment with sunitinib in patients with newly diagnosed glioblastoma. Clin Cancer Res. 2022;28(8):1595–1602. 35165100 10.1158/1078-0432.CCR-21-1933 PMC9365363

[26] Labots M, Pham TV, Honeywell RJ, et al Kinase inhibitor treatment of patients with advanced cancer results in high tumor drug concentrations and in specific alterations of the tumor phosphoproteome. Cancers (Basel). 2020;12(2):330. 32024067 10.3390/cancers12020330 PMC7072422

[27] Rovithi M, Gerritse SL, Honeywell RJ, et al Phase I dose-escalation study of once weekly or once every two weeks administration of high-dose sunitinib in patients with refractory solid tumors. J Clin Oncol. 2019;37(5):411–418. 30586316 10.1200/JCO.18.00725 PMC6368417

[28] Gerritse SL, Labots M, ter Heine R, et al High-dose intermittent treatment with the multikinase inhibitor sunitinib leads to high intra-tumor drug exposure in patients with advanced solid tumors. Cancers (Basel). 2022;14(24):6061. 36551546 10.3390/cancers14246061 PMC9775433

[29] Rovithi M, de Haas RR, Honeywell RJ, et al Alternative scheduling of pulsatile, high dose sunitinib efficiently suppresses tumor growth. J Exp Clin Cancer Res. 2016;35(1):138. 27604186 10.1186/s13046-016-0411-2 PMC5013589

[30] Gotink KJ, Broxterman HJ, Labots M, et al Lysosomal sequestration of sunitinib: A novel mechanism of drug resistance. Clin Cancer Res. 2011;17(23):7337–7346. 21980135 10.1158/1078-0432.CCR-11-1667 PMC4461037

[31] Ciwit BV . Castor electronic data capture Amsterdam; 2016.

[32] Wen PY, Macdonald DR, Reardon DA, et al Updated response assessment criteria for high-grade gliomas: Response Assessment in Neuro-Oncology working group. J Clin Oncol. 2010;28(11):1963–1972. 20231676 10.1200/JCO.2009.26.3541

[33] US Department of Health and Human Services . Common Terminology Criteria for Adverse Events (CTCAE); 2010. Accessed 01 July 2023. https://evs.nci.nih.gov/ftp1/CTCAE/CTCAE_4.03/CTCAE_4.03_2010-06-14_QuickReference_8.5x11.pdf

[34] Aaronson NK, Ahmedzai S, Bergman B, et al The European Organization for Research and Treatment of Cancer QLQ-C30: A quality-of-life instrument for use in international clinical trials in oncology. J Natl Cancer Inst. 1993;85(5):365–376. 8433390 10.1093/jnci/85.5.365

[35] Taphoorn MJ, Claassens L, Aaronson NK, et al An international validation study of the EORTC brain cancer module (EORTC QLQ-BN20) for assessing health-related quality of life and symptoms in brain cancer patients. Eur J Cancer. 2010;46(6):1033–1040. 20181476 10.1016/j.ejca.2010.01.012

[36] Osoba D, Aaronson NK, Muller M, et al The development and psychometric validation of a brain cancer quality-of-life questionnaire for use in combination with general cancer-specific questionnaires. Qual Life Res. 1996;5(1):139–150. 8901377 10.1007/BF00435979

[37] Fayers PM, Aaronson NK, Bjordal K, et al The EORTC QLQ-C30 scoring manual. 3rd edn. European Organisation for Research and Treatment of Cancer; 2001.

[38] Sarkaria JN, Hu LS, Parney IF, et al Is the blood–brain barrier really disrupted in all glioblastomas? A critical assessment of existing clinical data. Neuro Oncol. 2017;20(2):184–191. 10.1093/neuonc/nox175 PMC5777482 29016900

[39] Rajneet KO, Rajendar KM, William FE. Pharmacokinetic assessment of efflux transport in sunitinib distribution to the brain. J Pharmacol Exp Ther. 2013;347(3):755–764. 24113148 10.1124/jpet.113.208959 PMC3836310

[40] Tang SC, Lagas JS, Lankheet NAG, et al Brain accumulation of sunitinib is restricted by P-glycoprotein (ABCB1) and breast cancer resistance protein (ABCG2) and can be enhanced by oral elacridar and sunitinib coadministration. Int J Cancer. 2012;130(1):223–233. 21351087 10.1002/ijc.26000

[41] Eisenbarth D, Wang YA. Glioblastoma heterogeneity at single cell resolution. Oncogene. 2023;42:2155–2165. 37277603 10.1038/s41388-023-02738-y PMC10913075

[42] Yang Y, Li S, Wang Y, Zhao Y, Li Q. Protein tyrosine kinase inhibitor resistance in malignant tumors: Molecular mechanisms and future perspective. Signal Transduct Target Ther. 2022;7(1):329. 36115852 10.1038/s41392-022-01168-8 PMC9482625

[43] van Erp NP, Gelderblom H, Guchelaar H-J. Clinical pharmacokinetics of tyrosine kinase inhibitors. Cancer Treat Rev. 2009;35(8):692–706. 19733976 10.1016/j.ctrv.2009.08.004

[44] Pearson JRD, Cuzzubbo S, McArthur S, et al Immune escape in glioblastoma multiforme and the adaptation of immunotherapies for treatment. Front Immunol. 2020;11:582106. 33178210 10.3389/fimmu.2020.582106 PMC7594513

[45] Kim G, Ko YT. Small molecule tyrosine kinase inhibitors in glioblastoma. Arch Pharm Res. 2020;43(4):385–394. 32239429 10.1007/s12272-020-01232-3

[46] Wen PY, Stein A, van den Bent M, et al Dabrafenib plus trametinib in patients with BRAFV600E-mutant low-grade and high-grade glioma (ROAR): A multicentre, open-label, single-arm, phase 2, basket trial. Lancet Oncol. 2022;23(1):53–64. 34838156 10.1016/S1470-2045(21)00578-7

[47] Weller M, Le Rhun E. How did lomustine become standard of care in recurrent glioblastoma? Cancer Treat Rev. 2020;87:102029. 32408220 10.1016/j.ctrv.2020.102029

[48] Hegi ME, Diserens A-C, Gorlia T, et al MGMT gene silencing and benefit from temozolomide in glioblastoma. N Engl J Med. 2005;352(10):997–1003. 15758010 10.1056/NEJMoa043331

[49] Chahal M, Xu Y, Lesniak D, et al MGMT modulates glioblastoma angiogenesis and response to the tyrosine kinase inhibitor sunitinib. Neuro Oncol. 2010;12(8):822–833. 20179017 10.1093/neuonc/noq017 PMC2940678

[50] Faye MD, Easaw J, De Robles P, et al Phase II trial of concurrent sunitinib, temozolomide, and radiotherapy with adjuvant temozolomide for newly diagnosed MGMT unmethylated glioblastoma. Neurooncol Adv. 2023;5(1):vdad106. 10.1093/noajnl/vdad106 PMC10530294 37771465

[51] Cherny NI, Sullivan R, Dafni U, et al A standardised, generic, validated approach to stratify the magnitude of clinical benefit that can be anticipated from anti-cancer therapies: The European Society for Medical Oncology Magnitude of Clinical Benefit Scale (ESMO-MCBS). Ann Oncol. 2015;26(8):1547–1573. 26026162 10.1093/annonc/mdv249

[52] Cherny NI, Dafni U, Bogaerts J, et al ESMO-magnitude of clinical benefit scale version 1.1. Ann Oncol. 2017;28(10):2340–2366. 28945867 10.1093/annonc/mdx310

[53] Cherny NI, De Vries EGE, Dafni U, et al Comparative assessment of clinical benefit using the ESMO-magnitude of clinical benefit scale version 1.1 and the ASCO value framework net health benefit score. J Clin Oncol. 2019;37(4):336–349. 30707056 10.1200/JCO.18.00729

