# Supplementary material for: The STELLAR trial: a phase II/III randomized trial of high-dose, intermittent sunitinib in patients with recurrent glioblastoma
Source: Brain Commun. 2024 Jul 30;6(4):fcae241. doi: 10.1093/braincomms/fcae241 (PMC11303865; doi:10.1093/braincomms/fcae241)
Supplement: fcae241_Supplementary_Data [file fcae241_supplementary_data.pdf]

Supplementary Figure 1

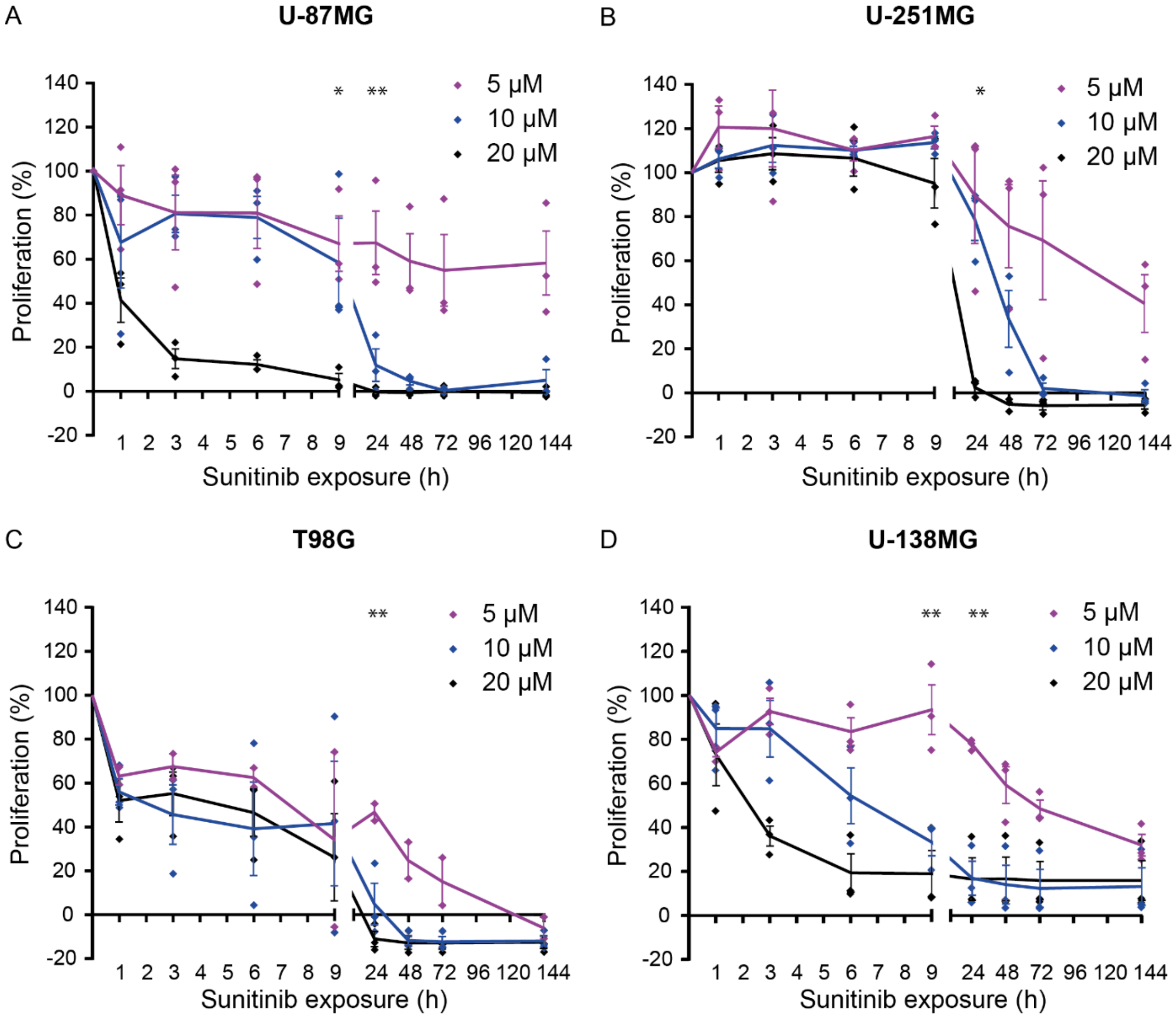

Supplementary Figure 1: High-dose, short-term sunitinib exposure of glioblastoma cell lines. **(A)** U-87MG **(B)** U-251MG **(C)** T98G **(D)** U-138MG. x-axes defines the time of sunitinib exposure in hours (h) and y-axes show percentage of proliferation compared to control untreated cells determined with an MTT assay. Different colours represent the sunitinib concentration cells were exposed to. All data points represent three independent biological replicates performed in triplicate (except 5 μM T98G). Erros bars indicate the standard error of the mean of the three biological replicates. At 9 and 24 hours, effect of exposure to 20 μM was compared to 5 μM using one-way ANOVA with Tukey's post-hoc test. * $p<0.05$ ** $p<0.01$. SUN: sunitinib.

Supplementary Figure 2

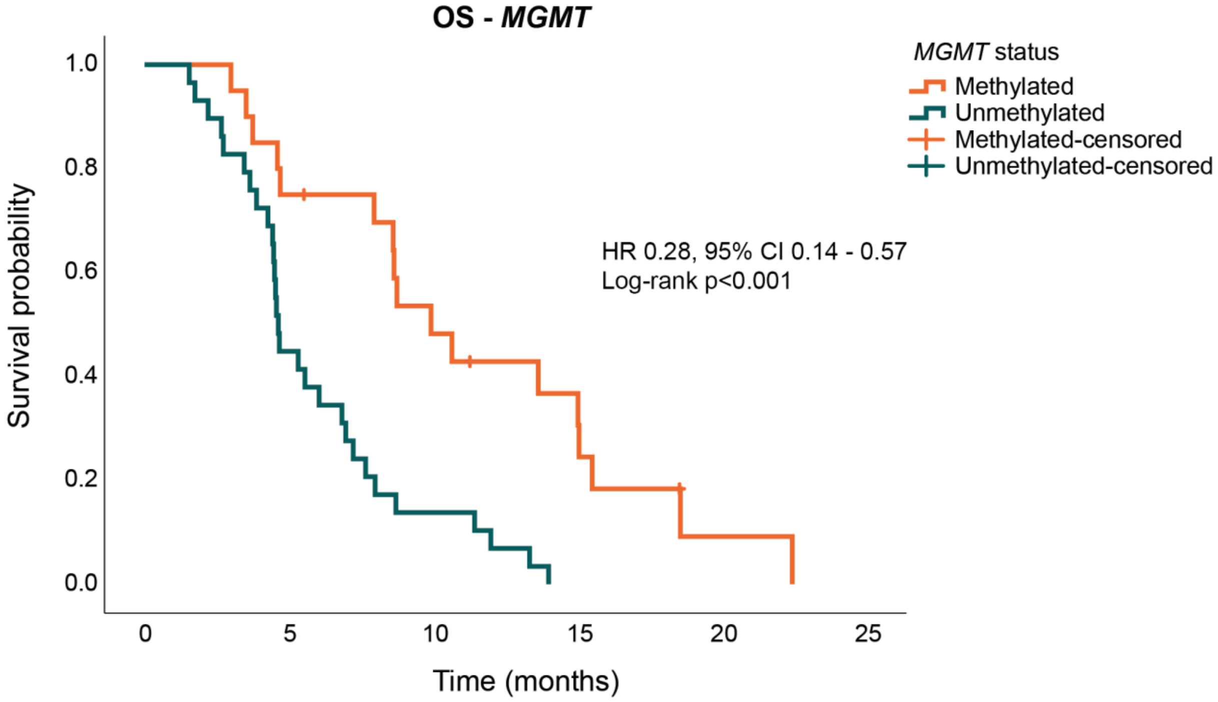

Supplementary Figure 2: *MGMT* status of all patients in relation to OS

Included in this graph are n=20 patients with methylated *MGMT* and n=29 patients with unmethylated *MGMT*. Abbreviations: *MGMT*, O^6-methylguanine-DNA methyl-transferase; HR, hazard ratio; OS, overall survival.

Supplementary Figure 3

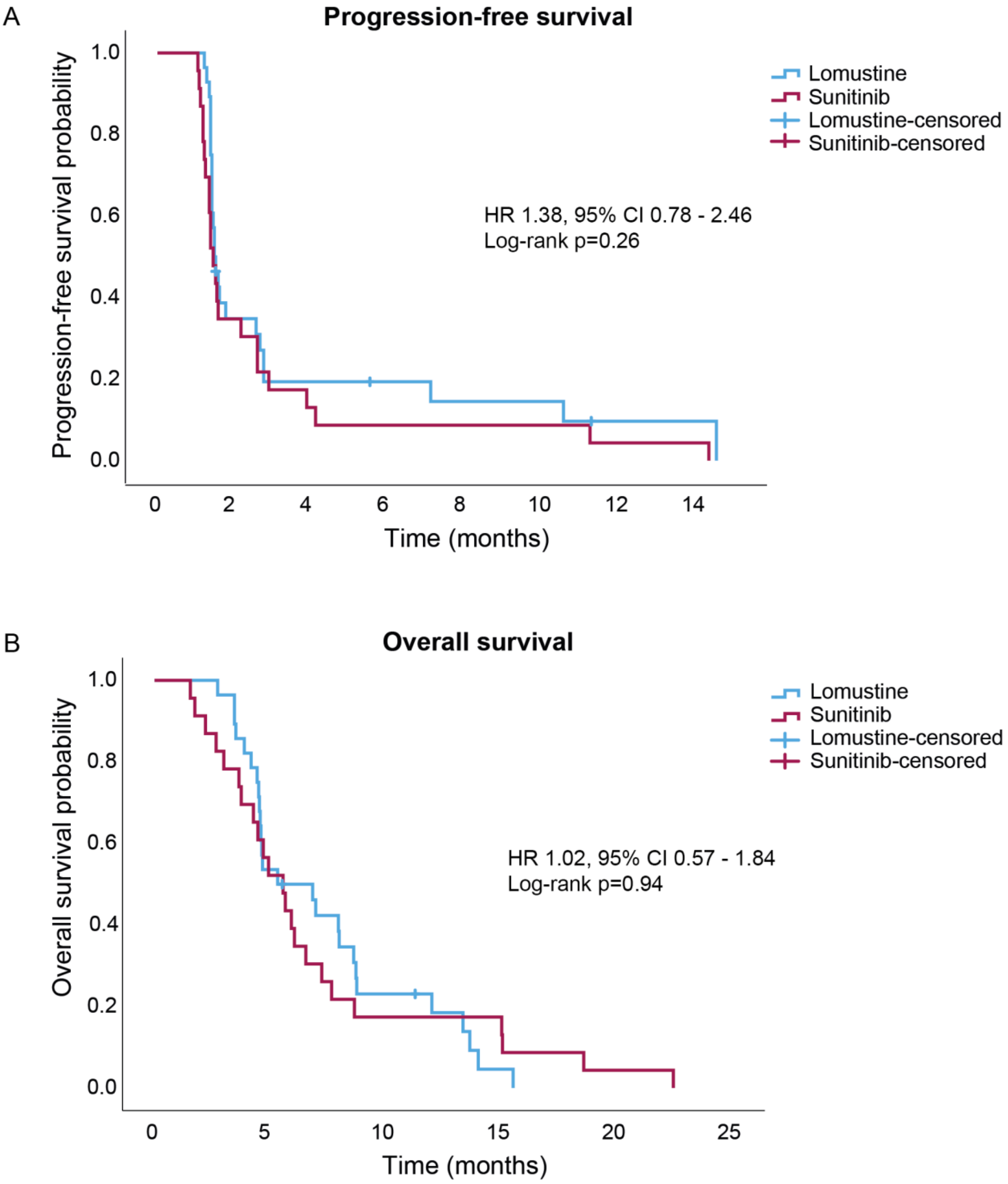

Supplementary Figure 3: exploratory analysis *IDH* mutation status

Progression-free survival (A) and overall survival (B) in an exploratory analysis which excluded patients with known *IDH* mutation. Analysis includes n=28 patients treated with lomustine and n=23 patients treated with high-dose intermittent sunitinib (300 mg Q1W or 700 mg Q2W). Abbreviations: HR, hazard ratio.

Supplementary Table 1. Best radiological response according to RANO criteria in part 1 and 2. Abbreviations: Q1W, weekly; Q2W, bi-weekly.

	Part 1		Part 2	
	**Sunitinib 300 mg Q1W (n=12)**	**Lomustine (n=14)**	**Sunitinib 700 mg Q2W (n=14)**	**Lomustine (n=15)**
**Objective response**	1 (8%)	1 (7%)	0 (0%)	0 (0%)
**Stable disease**	5 (42%)	5 (36%)	6 (43%)	5 (33%)
**Progressive disease**	6 (50%)	8 (57%)	8 (57%)	10 (67%)

Supplementary Table 2. Estimated marginal means EORTC QLQ-C30 and EORTC QLQ-BN20 per time point

	Baseline	Follow-up 1	Follow-up 2	Follow-up 3	Follow-up 4
**EORTC QLQ-C30 Summary score, mean score (standard error)**					
**Lomustine**	85,50 (2,56)<br>N=19	79,08 (2,67)<br>N=17	77,74 (3,67)<br>N=8	87,51 (4,98)<br>N=4	81,10 (5,67)<br>N=3
**Sunitinib**	88,77 (2,71)<br>N=17	84,00 (2,85)<br>N=15	86,50 (3,91)<br>N=7	92,44 (6,93)<br>N=2	85,64 (9,51)<br>N=1
**EORTC QLQ-C30 Physical functioning, mean score (standard error)**					
**Lomustine**	84,91 (4,38)<br>N=19	76,62 (4,53)<br>N=17	73,13 (5,98)<br>N=8	88,11 (7,89)<br>N=4	80,96 (8,91)<br>N=3
**Sunitinib**	89,02 (4,63)<br>N=17	87,47 (4,83)<br>N=15	86,17 (6,36)<br>N=7	88,13 (10,82)<br>N=2	95,34 (14,65)<br>N=1
**EORTC QLQ-BN20 visual disorders, mean score (standard error)**					
**Lomustine**	14,82 (5,47)<br>N=18	23,02 (5,62)<br>N=16	23,21 (6,85)<br>N=8	19,09 (8,65)<br>N=4	16,66 (9,63)<br>N=3
**Sunitinib**	12,42 (5,63)<br>N=17	17,89 (5,81)<br>N=15	8,60 (7,21)<br>N=7	10,09 (11,52)<br>N=2	8,50 (15,27)<br>N=1
**EORTC QLQ-BN20 motor dysfunction, mean score (standard error)**					
**Lomustine**	9,26 (3,96)<br>N=18	19,86 (4,08)<br>N=16	20,80 (5,12)<br>N=8	19,51 (6,61)<br>N=4	7,79 (7,41)<br>N=3
**Sunitinib**	7,84 (4,07)<br>N=17	10,53 (4,22)<br>N=15	9,08 (5,40)<br>N=7	2,53 (8,93)<br>N=2	10,39 (11,98)<br>N=1
**EORTC QLQ-BN20 communication deficit, mean score (standard error)**					
**Lomustine**	15,43 (4,95)<br>N=18	31,54 (5,11)<br>N=16	29,96 (6,41)<br>N=8	21,41 (8,27)<br>N=4	14,16 (9,27)<br>N=3
**Sunitinib**	7,19 (5,10)<br>N=17	12,86 (5,29)<br>N=15	6,55 (6,76)<br>N=7	4,31 (11,17)<br>N=2	1,88 (14,98)<br>N=1

Supplementary methods

Full inclusion and exclusion criteria

Inclusion criteria

In order to be eligible to participate in this study, a subject must meet all of the following criteria:

1. Signed (by the patient or legally acceptable representative) and dated Informed Consent Form.
2. Histologically confirmed de novo or secondary glioblastoma with unequivocal first progression, at least 3 months off radiotherapy.
3. No more than one line of chemotherapy (concurrent and adjuvant temozolomide based chemotherapy including in combination with another investigational agent is considered one line of chemotherapy). Chemotherapy must have been completed at least 4 weeks prior to randomization.
4. Patients may have undergone surgery for recurrence. If operated, residual and measurable disease after surgery is not required but surgery must have confirmed the recurrence.
5. No radiotherapy, stereotactic radiosurgery or brachytherapy as treatment for recurrence.
6. Patients must have a Karnofsky Performance Score ≥ 70%
7. Patients need to have adequate hematological, renal and hepatic function as assessed by the following laboratory requirements to be conducted within seven days prior to start study treatment:
   a. Hemoglobin ≥ 7.0 mmol/L
   b. Absolute neutrophil count (ANC) ≥ 1.5 x 10^9/L
   c. Platelet count ≥ 100 x 10^9/L
   d. ALAT and ASAT ≤ 2.5 x ULN
   e. Serum creatinine eGFR ≥ 50 ml/min
   f. Albumin ≥ 25 g/L
8. Age ≥ 18 years
9. Male and female patients with reproductive potential must use an approved contraceptive method during and for three months after discontinuation of study treatment.
10. Patients must be able to swallow oral medication.

Exclusion criteria

A potential subject who meets any of the following criteria will be excluded from participation in this study:

1. Evidence of a significant uncontrolled concomitant disease, such as cardiovascular disease (including stroke, New York Heart Association Class III or IV cardiac disease or myocardial infarction within 6 months prior to screening, unstable arrhythmia, clinically significant valvular heart disease and unstable angina); nervous system, pulmonary (including obstructive pulmonary disease and history of symptomatic bronchospasm), renal, hepatic, endocrine, or gastrointestinal disorders; or a serious non-healing wound or fracture.
2. Second primary malignancy within the past 5 years with the exception of adequately treated in situ carcinoma of any organ or basal cell carcinoma of the skin.

3. Prior radiotherapy in the abdomen or in the lungs or in more than 3 vertebrae in the spine (Less than 3 vertebrae are considered a small radiation field and eligibility will be decided on an individual basis from the principal investigator).
4. Poorly controlled hypertension despite adequate blood pressure medication. Blood pressure must be ≤ 160/95 mmHg at the time of screening on a stable antihypertensive regimen. Blood pressure must be stable on at least 2 separate measurements.
5. Known active bacterial, viral, fungal, mycobacterial, or other infection (including HIV and atypical mycobacterial disease, but excluding fungal infection of the nail beds).
6. Initial MR-scan (at baseline of study) of the brain showing intratumoral hemorrhage, except for stable post-operative grade 1 hemorrhage.
7. Known hypersensitivity to sunitinib or to its excipients.
8. Presence of any significant central nervous system or psychiatric disorder(s) that would interfere with the patient's compliance.
9. Use of full-dose oral or parenteral anticoagulants or thrombolytic agent for therapeutic (as opposed to prophylactic) purposes.
10. Use of strong hepatic enzyme-inducing antiepileptic drugs, such as carbamazepine, phenobarbital and phenytoin. If a patient uses one or more of these specific antiepileptic drugs, they must switch to an antiepileptic drug that does not interact with cytochromeP450 (CYP450) liver enzymes, such as levetiracetam, prior to the start of study treatment.
11. Drug or alcohol abuse.
12. Females who are pregnant or breast-feeding.
13. Any evidence of a disease or condition that might affect compliance with the protocol or interpretation of the study results or render the patient at high risk from treatment complications.
14. Unwillingness or inability to comply with study and follow-up procedures.
15. Clinically significant history of liver disease, including viral or other hepatitis, current alcohol abuse, or cirrhosis.

***In vitro* high-dose short-term exposure experiments**

The established cell lines U-87MG, U-251MG, U-138MG and T98-G GBM cell lines were acquired from the ATCC and cultured in Dulbecco's Modified Eagle's Medium supplemented with 5% fetal bovine serum (FBS) at 37°C 5% CO_2. Two-thousand cells per well were seeded in a 96-well plate. Twenty-four hours after seeding, cells were exposed to sunitinib-medium solution with final drug concentrations of 5 µM, 10 µM and 20 µM. Medium was removed after 1, 3, 6, 9, 24, 48, 72 or 144 hours of treatment, cells were washed with PBS and fresh drug-free medium was added. All experiments were performed in triplicate and repeated as three independent biological replicates. 144 hours after first exposure viability was assessed with the use of tetrazolium 3-(4,5-dimethylthiazol-2-yl)-2,5-diphenyltetrazolium bromide (MTT) as described previously.[1,2] In short, medium was removed and 50 µl MTT (5 mg/ml, diluted 1:11 in cell culture medium) indicator dye was added to the wells. After 90 minutes incubation at 37 °C 150 µl DMSO was added and absorption was measured after 20 minutes at 540nm in a microplate reader (Spectra Fluor Tecan, Salzburg, Austria). Untreated cells cultured in control medium

were used as a reference value. Formula for calculation of cell proliferation was used as described previously.[1]

References

1. Rovithi M, de Haas RR, Honeywell RJ, et al. Alternative scheduling of pulsatile, high dose sunitinib efficiently suppresses tumor growth. *J Exp Clin Cancer Res*. Sep 7 2016;35(1):138. doi:10.1186/s13046-016-0411-2

2. Gotink KJ, Broxterman HJ, Labots M, et al. Lysosomal sequestration of sunitinib: a novel mechanism of drug resistance. *Clin Cancer Res*. Dec 1 2011;17(23):7337-46. doi:10.1158/1078-0432.Ccr-11-1667
